# The Main Vectors of the US Foreign Policy Strategy in Central Asia

**DOI:** 10.1134/S1019331622130056

**Published:** 2022-12-23

**Authors:** A. A. Kokoshin, Z. A. Kokoshina

**Affiliations:** 1 Higher School of Economics (HSE), Moscow, Russia; 2 Moscow State University, Moscow, Russia; 3 Institute of Sociology, Federal Center of Theoretical and Applied Sociology, Russian Academy of Sciences, Moscow, Russia

**Keywords:** Central Asia, US strategy, Kazakhstan, Uzbekistan, Kyrgyzstan, energy resources, geopolitical positions, political and ideological impact, social and humanitarian sphere, nongovernment organizations

## Abstract

The main vectors of the US foreign policy strategy regarding the countries of Central Asia (CA) after the collapse of the Soviet Union are analyzed. It is noted that there are three main vectors in the US strategy in Central Asia: geopolitical, economic, and political−ideological, and humanitarian, which in many cases are closely intertwined with each other. Kazakhstan and Uzbekistan are of the main interest to the United States there. The authors draw attention to the fact that the US strategy in this region has an obvious anti-Russian and anti-Chinese orientation, which has intensified during the crisis of 2021‒2022, caused by the refusal of the United States and its allies to meet the legitimate claims of the Russian Federation adequately to ensure its security.

## INTRODUCTION

The three main vectors of the US foreign policy strategy in Central Asia (CA) can be defined as follows:

*Geopolitical vector,* which manifests itself in diplomatic activity and efforts in the field of military and armaments cooperation.

*The economic vector* is mainly expressed in the interest of the American state and business in the maximum possible control over the very significant energy resources of Central Asia, primarily Kazakhstan with its deposits of natural gas, oil, and uranium.

With regard to *the political-ideological, and humanitarian vector*, we primarily mean the US long-term plan to “democratize” CA countries along the Western lines.

All these vectors have an obvious anti-Russian (and anti-Chinese) bias.

In geopolitical terms, CA is dominated by Russia in the first place, which has very important bilateral relations in the political−military sphere, as well as in the field of security with each of the Central Asian countries, former Soviet republics. The Russian Federation prevails in armaments cooperation with these countries and provides them with considerable assistance in training personnel for law enforcement agencies. There are important military facilities of the Russian Federation on the territory of the Central Asian countries (especially in Kazakhstan). In addition to bilateral ties with the Central Asian countries, in the geopolitical vector Russia has formats such as the Collective Security Treaty Organization (CSTO) (with the participation of Kazakhstan, Tajikistan, and Kyrgyzstan) and the Shanghai Cooperation Organization (SCO) (which, in addition to those mentioned, also includes Uzbekistan). Evidence of Russia’s significant influence in Central Asia was the successfully conducted CSTO peacekeeping operation for a short time at the request of the leadership of Kazakhstan in January 2022. As is known, the Russian contingent played a leading role in this operation.

As for the economic vector, China’s presence in Central Asia is becoming more and more noticeable, both directly through the state line and through private companies that are massively supported by the state. However, as before, the main role in this area is played by the economic relations of the Central Asian countries with the Russian Federation, both bilateral and within the framework of the Russia-led Eurasian Economic Union (in which Central Asia is represented by Kazakhstan, Tajikistan, and Kyrgyzstan).

Russia manages to hold significant positions in this region in the political, ideological, and humanitarian areas as well despite the large-scale efforts of US government and especially nongovernment organizations.

Among the most important instruments of US influence on the Central Asian countries is the political format C5 + 1 (CA5 + USA), created by Washington in 2015. It also provides for regular meetings of the foreign ministers of the Central Asian countries and the US Secretary of State [Yunyushkina et al., 2021, p. 46].

In February 2020, the US Strategy for Central Asia 2019−2025, adopted by the Trump Administration, was published. The presentation of this document took place in the Heritage research and advocacy center, close to the Republicans.^1^ There is much evidence that the provisions of this strategy retain their significance under the Biden Administration.

## GEOPOLITICAL VECTOR

This vector of the US foreign policy strategy in Central Asia was initially associated with the desire of the United States to impede the reintegration efforts of Russia in the post-Soviet space, limiting Russia’s influence in Central Asia in every possible way. As China’s economic and military power and foreign policy influence grow, the United States increasingly faces the task of preventing the further strengthening of the PRC in this region, which is becoming increasingly significant and multifaceted in terms of mutual trade and Chinese investment [Ryazantsev et al., 2019, pp. 20‒35]. This is part of the growing global confrontation between the United States and China, which is becoming more and more acute and large-scale.

The setting for counteracting reintegration processes with the leading role of Russia is expressed in the formula of ensuring independence and “individual sovereignty.” The aforementioned US strategy in Central Asia aims to “support and strengthen the sovereignty and independence of the Central Asian States, individually and as a region,” which should be carried out “with consistent US engagement on economic, energy, security, democracy, and governance issues” of the Central Asian states. As follows from this document, the United States seeks to increase its influence in Central Asia under the pretext of helping to “reduce terrorist threats” for the countries of this region. This is explained as a bilateral activity to understand, identify, prevent, and counter “violent extremism.” The document also envisages joint efforts of the United States and the Central Asian countries to “return, rehabilitate, and reintegrate foreign terrorist fighters and their families into society” and to “strengthen the capacity of law enforcement and security services to protect borders and interdict the movement of terrorists and trafficking into and across Central Asia.”^2^

For a long time, the geopolitical direction of the US foreign policy strategy was closely connected with the military actions of the United States and its allies and partners in Afghanistan after the acts of “megaterror” on September 11, 2001, as part of Operation Enduring Freedom. Since the start of this operation, the Central Asian countries have been viewed by Washington mainly as a “gateway” to Afghanistan, where the United States and its allies conducted rather large-scale military operations against Al-Qaeda and the Taliban [Rumer et al., 2016]. The military actions of the United States and its allies in Afghanistan continued with varying degrees of intensity until August 2021, when everything ended in the virtual flight of the United States from this country and the collapse of the pro-Western regime in the face of the military and political successes of the Taliban movement.

Immediately after 9/11, the United States began negotiating agreements with Uzbekistan and Kyrgyzstan to use existing bases and deploy troops in support of the war in Afghanistan. It was about the Manas airfield in Kyrgyzstan and the Karshi-Khanabad base in Uzbekistan, which were supposed to perform the functions of supplying and supporting the actions of the United States and its allies in Afghanistan.^3^

Soon the United States signed a number of agreements not only on military bases but also on the use of airspace and the logistics of its contingents at these bases in Central Asia [Cooley, 2021].

A. Cooley believes that the then President of Uzbekistan, Islam Karimov, saw his new partnership with the United States as an opportunity to give legitimacy to his domestic line against Islamist extremist militants [Cooley, 2021].

In May 2005, against the backdrop of antigovernment protests, Islamists attempted to seize power in the city of Andijan; the attempt was severely suppressed by Karimov and his security forces. After such actions of the Uzbek authorities in Andijan, Washington announced the need for an independent investigation of the events at the OSCE level. This caused an extremely negative reaction from Karimov, who quickly demanded that the American presence on the territory of Uzbekistan be curtailed by December 2005. The United States was compelled to satisfy this demand. As a result, Washington lost a very important military facility. Part of the personnel of the Karshi-Khanabad base was transferred to Kyrgyzstan, to the Manas international airport.

Relations between the United States and Uzbekistan deteriorated over more than a decade.

An agreement with Kyrgyzstan on the provision of a part of the Manas civil airport for basing military personnel and military equipment participating in the operation in Afghanistan was concluded on December 4, 2001, for a year with the possibility of further automatic prolongation. Bishkek also allowed the passage of American aircraft through its airspace [Shukurov, 2022]. According to Sh. Z. Shukurov, there were no objections from Russia because at that time “the United States was perceived as a victim of terrorist acts” [Shukurov, 2022].

Bishkek also agreed to base the forces and means of 11 other countries in addition to the United States in Manas; in general, about 1000 military personnel and several military transport aircraft and tanker aircraft were constantly present at this airbase.^4^

Ultimately, largely under the influence of Russia, the US base at Manas was closed in 2014. Thus ended the permanent military presence of the United States in the region.

Returning to US relations with Uzbekistan, we can note that they began to improve after the death of Karimov, with the coming to power of Sh.M. Mirziyoyev, who visited the United States in September 2017 to attend the 72nd session of the UN General Assembly and met there with the President of the United States D. Trump, as well as with the leadership of a number of large American companies. These meetings resulted in agreements on investments in the economy of Uzbekistan in the amount of $2.6 bln [Izteleulova and Lapenko, 2021].

In May 2018, Mirziyoyev’s first official visit to Washington took place. As a result of his negotiations with Trump, a Joint Statement of the heads of both states—Uzbekistan and the United States: The Start of a New Era of Strategic Partnership—was adopted. Several agreements were signed aimed at developing ties between the two countries in various fields. Among them is a five-year plan for military cooperation.

As a result of Mirziyoyev’s visit to Washington in 2018, the number of joint military exercises between Uzbekistan and the United States has increased sharply, their main goal being to strengthen cooperation in the field of security and improve interaction between the two countries. In January 2019, servicemen of the special operations forces of the Ministry of Defense of Uzbekistan took part in the Southern Strike joint exercise at the military base Camp Shelby (Mississippi). On September 10 of the same year, American and Uzbek pilots conducted joint exercises in the Chirchiq garrison near Tashkent [Gegelashvili and Modnikova, 2021]. In March 2020, the joint exercises of the United States and Uzbekistan were also held on the territory of Uzbekistan.

On a rather significant scale, training of the military personnel of Uzbekistan is carried out in the United States; American military instructors teach at military educational institutions in Uzbekistan [Ponomarev, 2020].

As for Kazakhstan, the United States has made significant political and military efforts in this direction. Back in 2000, before the start of Operation Enduring Freedom, with the participation of the United States and other NATO members, a KAZBAT battalion was formed in Kazakhstan on the basis of the third air assault battalion of the Kapshagai air assault brigade according to NATO standards and to solve joint tasks with NATO.^5^ This battalion was involved in two projects: sending a unit to Iraq and conducting the annual Steppe Eagle peacekeeping exercises in Kazakhstan together with contingents of NATO countries. In August 2003, Kazakhstan sent KAZBAT troops to Iraq as part of the US-led coalition [Stein, 2018]. In December 2006, KAZBAT was transformed into KAZBRIG (two more battalions were added to it, also armed and equipped according to NATO standards) [Stein, 2018].

In 2003−2019, within the framework of military cooperation between the United States and Kazakhstan, Steppe Eagle joint exercises were regularly held with the participation of contingents from other countries. In 2020 and 2021, during the coronavirus pandemic, the exercises were not conducted.

Military specialists from the United States and other NATO countries are actively working in several training centers for personnel of the armed forces of Kazakhstan.^6^

Of all the Central Asian countries, Kazakhstan is the main recipient of US military assistance.^7^

State Duma Deputy G. Onishchenko drew attention to the functioning of the laboratory built by the US Department of Defense near Almaty, which “develops military biological formulations.”^8^ There are objects of this kind in many other countries of the world (during a special military operation of the Russian Armed Forces, they were discovered in Ukraine). Countries hosting such American facilities receive “official assistance” in organizing the accounting and safe storage of microbial collections, in reconstructing laboratory facilities, and in training specialists. The United States seeks to obtain complete control over the sanitary and epidemiological situation and research in the respective country. The United States seeks to replenish its collections of biomaterials, to study the susceptibility of residents to various diseases and their treatments, and to test innovative drugs on the local population.^9^

Russia has repeatedly noted that such activities are a violation by the United States of the 1972 Convention on the Prohibition of the Development, Production, and Stockpiling of Bacteriological (Biological) and Toxin Weapons and on Their Destruction.

In May 2021, the well-informed American *Wall Street Journal* reported that Washington was considering deployment options for its troops that the United States was going to withdraw from Afghanistan, with Uzbekistan and Tajikistan as a priority. According to the sources who provided this information to the newspaper, deployment in these countries will allow the United States to respond quickly to what is happening in Afghanistan since they share common borders with the country.^10^ Later, a statement from the Uzbek Ministry of Defense said that the United States had approached Uzbekistan. However, it was stated that the appearance of US military facilities on the territory of the country is out of the question.^11^

In his speech on March 31, 2022, at a meeting of Afghanistan’s neighbors (Russia, China, Iran, Pakistan, Tajikistan, Turkmenistan, and Uzbekistan) in China, Russian Minister of Foreign Affairs S.V. Lavrov said that “Moscow considers unacceptable the deployment of the military infrastructure of NATO, the United States, or the Afghans working for them in neighboring countries, primarily in the states of Central Asia.”^12^

## ECONOMIC VECTOR

Within this vector, the United States and American business have mostly been interested in the raw materials of the Central Asian countries, primarily Kazakhstani hydrocarbons. In some cases, the geopolitical use of these resources in the interests of the United States and its allies is being considered.

In 2019, Kazakhstan ranked 12th in the world in terms of proven reserves of oil and gas condensate with a production volume of 90.5 million tons of oil, exporting 72.4% of the liquid fuel produced. In terms of proven reserves of natural gas, Kazakhstan in 2019 ranked 22nd in the world; gas production in the same year reached 56.4 billion cubic meters. Kazakhstan also ranks 8th in the world in coal production. The total volume of recoverable reserves of fuel resources (oil, gas, coal, and uranium) of Kazakhstan is estimated at about 32 billion tons of oil equivalent (TOE) [Zhanbulatova et al., 2021, p. 21]. As noted on the website of the Energy Information Administration of the US Department of Energy, Kazakhstan “has the second largest oil reserves and the second largest oil production after Russia among the former Soviet republics.”^13^

Kazakhstan, according to American data, has 12% of the world’s uranium resources; since 2009, it has been the world leader in uranium production, up to 43% of world production.^14^ In 1997, the President of Kazakhstan, N. Nazarbayev, nationalized a large part of the uranium industry, creating Kazatomprom (KAP). This state-owned holding mines uranium in the country together with the Canadian company Uranium One, which has been 100% owned by Russian Rosatom since 2013.^15^ According to some reports, a Kazakh−Chinese joint venture also operates in this sphere.

Since 1993, most investment in Kazakhstan’s oil and gas sector has come from the United States.^16^ Major American energy companies such as Chevron and Exxon-Mobile are among the key players in Kazakhstan’s oil sector.^17^

The United States enjoys a significant presence in many other sectors of the Kazakh economy.

A significant strengthening of the US position in Kazakhstan was the result of Nazarbayev’s “multi-vector policy”—with all his outward demonstration of special loyalty to Russia and initiatives in the development of Eurasian integration. According to many estimates, the positions of the Russian Federation in Kazakhstan and in Central Asia as a whole remain predominant in comparison with the United States and other Western countries. Thus, Head of the Russian Ministry of Foreign Affairs Lavrov stressed that “the volume of economic ties now being built between the United States and the European Union and Central Asia is incomparable with our economic interpenetration, but the goal set [by the West] is unambiguous—to weaken our ties with our allies and strategic partners in every possible way.”^18^

Many in the United States and European Union take a stand for making gas from Turkmenistan an alternative to Russian gas. A trans-Caspian pipeline is proposed for the European market (then through Azerbaijan, Georgia, and beyond). It is hoped that this will be facilitated by the agreement concluded in 2021 (after lengthy and difficult negotiations) between Azerbaijan and Turkmenistan on the joint exploitation of gas fields along the sea border in the Caspian Sea.^19^

## THE POLITICAL−IDEOLOGICAL AND HUMANITARIAN VECTOR

The end of the Cold War not only failed to weaken but even strengthened this vector in US foreign policy as a whole, which was also reflected in the American strategy in relation to the Central Asian countries.

Since the Clinton Administration (the 1990s), the United States has been guided by the promotion of Western “democratic values” to Central Asia, which has been stably maintained by both government and nongovernment actors for many years.

The US strategic line for active political and ideological influence on the Central Asian countries in the above-mentioned US Strategy for Central Asia 2019‒2025 is expressed in following formula: “strong democratic institutions, rule of law, and respect for human rights.”^20^ At the same time, according to V.A. Ponomarev, in recent years Washington and American NGOs have shown respect for the “special path of development” of the Central Asian countries, and the impact on these countries in the political, ideological, and humanitarian spheres has become more flexible. However, it remains constant and purposeful and is expressed in pressure on the countries of the region with reminders of the need to liberalize national legislation regulating the electoral process, create “free media,” “a multiparty political system,” etc. [Ponomarev, 2020, p. 453].

There is much evidence that American organizations seek to present the domestic and foreign policy of the Russian Federation in an unfavorable light to the population of the Central Asian countries, especially in the context of the aggravation by the United States and the “collective West” of relations with the Russian Federation in connection with the crisis associated with Ukraine. Many attacks were made against the actions of the CSTO to help stabilize the situation in Kazakhstan in January 2022.

Washington is exerting various kinds of pressure on the Central Asian countries so that they do not act bypassing Western sanctions against Russia, which in the aggregate are actually an economic war against the Russian Federation.

According to a number of estimates, over the past 30 years, the United States has allocated about $9 billion in state aid to the Central Asian countries to implement “democratic reforms,” “maintain social and economic growth,” and to ensure security and humanitarian purposes [Yunyushkina et al., 2021, p. 43]. At the same time, many Western and Asian allies and partners of the United States are acting in this area in a similar spirit, demonstrating the existence of a “value delimitation” of the “collective West” with Russia and China [Pantin, 2021, pp. 8‒15]. The US government and nongovernment organizations have very significant financial resources and also attract funds from large American businesses [Velikaya, 2019, pp. 16‒18].

In the Central Asian countries, there are US government organizations such as the Agency for International Development and the US Information Agency. As for the Peace Corps, out of the five countries in the region, it operates only in Kyrgyzstan. The activities of the Peace Corps were terminated in 2011 in Kazakhstan and in 2012 in Turkmenistan. The United States hopes to restore its presence in Uzbekistan, where it operated in 1992−2005. The only Central Asian country where the Peace Corps has not yet begun to operate is Tajikistan [Velikaya, 2019, p. 18].

Political and propaganda activities in the Central Asian countries are also carried out directly by the American embassies in these countries. They are also represented in the most popular social networks of the post-Soviet space. Radio Liberty, an information tool of the US foreign policy strategy, is actively working in all languages of this region; there is also an online version of the Voice of America in Uzbek and Persian [Bakhirev, 2018, p. 35].

As for American nongovernment organizations in Central Asia, their number is especially large in Kazakhstan. American NGOs such as Azattyk (branches of Radio Liberty, United States), Present Time (subsidiary of Liberty), Eurasia, and many others are active in Central Asia. Notable is the activity of the National Endowment for Democracy. These organizations are also characterized by an anti-Chinese orientation.^21^

The Soros Foundation—Kazakhstan activities occupy a prominent place. According to the official reporting of this fund, it spent about $100 million in Kazakhstan from 1995 to 2020.^22^ As D. Rodionov notes, for a long time this fund has been engaged particularly in the formation of a negative attitude of Kazakh society to the joint operation of the Baikonur cosmodrome with Russia.^23^

In the social and humanitarian sphere, in addition to the Eurasia Foundation, the Council for International Research and Scientific Exchanges (IREX) is actively operating in Central Asia, implementing projects to develop education, in which government institutions and relevant ministries act as partners [Silakov, 2021, p. 116].

As V. Komleva notes, foreign financing of the activities of public organizations in Uzbekistan and Tajikistan is under the close attention of the state. In the Republic of Kazakhstan, since 2019, legislation has been changing toward strengthening accounting and control over foreign investments in NGOs [Komleva, 2021, p. 5].

There are three significant universities in the region under strong and in many ways direct influence of the United States: American University of Central Asia (AUCA, Bishkek), Kazakh−American University (KAU, Almaty), and Kazakhstan Institute of the World Economy and Entrepreneurship (KIMEP, Almaty). Note that the American University in Bishkek is not accountable to the Ministry of Education and Science of Kyrgyzstan; it has an American board of trustees. At Nazarbayev University, one of the leading universities in Kazakhstan, just like at KIMEP, teaching is conducted in English. Among the influential American humanitarian structures in Central Asia in the field of science and education is the American Central Asian Educational Foundation [Velikaya, 2019].

The US Strategy for Central Asia 2019−2025 notes that the American University in Bishkek is “a growing hub for the region’s best young minds to earn US degrees, gain in-demand business skills, and create life-long regional affiliations.”^24^

US government and nongovernment organizations in every possible way encourage the visits of Central Asian residents to the United States and large-scale study of the English language. Central Asians have made about 1.4 million visits to the United States, according to the State Department. Since independence by the Central Asian countries, over 40 000 students, professionals, and government officials from these countries have received US funding “to visit the United States for professional development opportunities.”^25^

## CONCLUSIONS

For many years, one of the most important tasks for the United States has been to counter the reintegration processes in the post-Soviet space under the leadership of Russia. The task of maximally weakening Russia’s positions in Central Asia has become even more significant for Washington in the context of the acute and deep crisis in relations between the “collective West” and the Russian Federation in 2021−2022, caused by the refusal of the United States and its allies to satisfy Russia’s legitimate claims to ensure its security.

The scale of US efforts in the Central Asian region as a whole, often coinciding with the efforts of American allies and partners in this area, is very significant. At the same time, in addition to US government agencies, numerous nongovernment organizations, and US private businesses, which have considerable financial resources, bear a very significant burden.

These efforts of the “collective West” run into deep and long-term mutual interests of the Central Asian countries, on the one hand, and the Russian Federation, on the other. An obstacle to the growth of US influence in Central Asia in the economic area is the growth of China’s economic presence in this region.

Central Asia is an area of vital strategic interests for Russia, including the most important national security interests of our country. This set and continues to set the task of the most active opposition to the US foreign policy strategy in Central Asia in all three areas discussed above, using for this both bilateral and multilateral mechanisms of cooperation between Russia and the Central Asian countries. At the same time, it is necessary to consider the specifics of each of the Central Asian countries; their traditions; culture (including the role of Islam); the peculiarities of the political system; and the nature of their activities within the framework of the CSTO, the CIS, the EAEU, and the SCO.

In particular, more attention should be paid to the activities of government and nongovernment Russian organizations in the political, ideological, and humanitarian spheres with account for the specifics of the conditions of the intense information war being waged against the Russian Federation by the United States and its allies in the Central Asian direction.

## Footnotes

1 Trump administration launches Central Asia strategy at Heritage (2020). https://www.heritage.org/asia/impact/trump-administration-launches-central-asia-strategy-heritage. Cited January 19, 2022.

2 United States strategy for Central Asia 2019‒2025: Advancing sovereignty and economic prosperity (overview) (2020), US Department of State, February 5. https://www.state.gov/united-states-strategy-for-central%20-asia-2019-2025-advancing-sovereignty-and-economic-prosperity/. Cited March 11, 2022.

3 United States foreign policy in the states of Central Asia (2002), The Brookings Institution, November 12. https://www.brookings.edu/events/united-states-foreign-policy-in-the-states-of-central-asia/. Cited May 12, 2022.

4 Manas air base on the territory of Kyrgyzstan (2013), ITAR TASS, November 14. https://tass.ru/info/751517?utm_source= yandex.ru&utm_medium=organic&utm_campaign=yandex.ru&utm_ referrer=yandex.ru. Cited January 21, 2022.

5 Popov, D. (2013) KAZBRIG as an indicator of the military-political rapprochement between Kazakhstan and the USA, RISS, December 23. https://riss.ru/article/7713/?. Cited January 23, 2022.

6 Mukhin, V. (2021) NATO forces are waiting in Nur-Sultan in July, Nezavisimaya gazeta, May 20. https://www.ng.ru/armies/2021-05-20/1_8153_nato.html?. Cited January 23, 2022.

7 Umarov, T. (2021) Everyone’s friend: Will Russia and China be able to force the US out of Kazakhstan, Carnegie Moscow Center Carnegie Endowment for International Peace, July 27. https://carnegie.ru/commentary/84968. Cited January 23, 2022.

8 G. Onishchenko: US biolaboratories in the CIS countries are dangerous—COVID has shown what viruses are capable of. (2021), Internet portal of the CIS, May 19. https://e-cis.info/news/566/92233/. Cited January 23, 2022.

9 Milov, K. (2021) Violation by the United States of America of the Conventions on the Prohibition of Chemical and Biological Weapons, *Foreign Military Review*, No. 6, 34. https://zvo.ric.mil.ru/Nomera/2/. Cited January 23, 2022.

10 Salama, V. and Gordon, L. (2021) Afghan pullout leaves U.S. looking for other places to station its troops, *Wall Street Journal*, May 8. https://www.wsj.com/articles/afghan-pullout-leaves-u-s-looking-for-other-places-to-station-its-troops-11620482659?mod= searchresults_pos3&page=1. Cited March 11, 2022.

11 Belen’kaya, M. and Krivosheev, K. (2021) Aid base extended to Washington, Kommersant, July 17. https://www.kommersant.ru/doc/4907714. Cited February 17, 2022.

12 Speech by the Minister of Foreign Affairs of the Russian Federation S.V. Lavrov during the plenary session at a meeting in the format of the neighboring countries of Afghanistan (Russia, China, Iran, Pakistan, Tajikistan, Turkmenistan, Uzbekistan), Website of the Russian Foreign Ministry, March 30 (2022). https://www.mid.ru/ru/foreign_policy/rso/nato/1807302/?lang=ru. Cited April 3, 2022.

13 Background reference: Kazakhstan (2019) U.S. Energy Information Administration, January 7. https://www.eia.gov/international/content/analysis/countries_long/Kazakhstan/background.htm. Cited March 11, 2022.

14 Central Asia: Background and US relations, Congressional Research Service, September 24 (2021). https://crsreports.congress.gov/product/pdf/R/R46924. Cited March 11, 2022.

15 Nikiforov, O. (2022) Kazakh uranium factor in political protests, *Nezavisimaya gazeta,* February 7. https://www.ng.ru/ng_energiya/2022-02-07/9_8365_factor.html. Cited April 23, 2022.

16 Time to share: Who is fighting for the energy resources of Kazakhstan (2022) RIA Novosti, January 15. https://ria.ru/20220115/kazakhstan-1767874161.html. Cited April 5, 2022.

17 What does the US mean for Kazakhstan? Figures and facts (2019), Forbes Kazakhstan, September 25. https://forbes.kz/life/opinion/chto_znachit_ssha_dlya_kazahstana_tsifryi_i_faktyi?. Cited April 5, 2022.

18 The countries of Central Asia and the US noted the success of the C5 + 1 format (2021), ITAR TASS, May 4. https://tass.ru/mezhdunarodnaya-panorama/11307235?utm_source=yandex.ru&utm_medium=organic&utm_campaign=yandex.ru&utm_ referrer=yandex.ru. Cited March 11, 2022.

19 Central Asia: Background and US Relations (2021) Congressional Research Service, September 24. https://crsreports.congress.gov/product/pdf/R/R46924. Cited March 11, 2022.

20 United States strategy for Central Asia 2019‒2025: Advancing sovereignty and economic prosperity (overview), U.S. Department of State, February 5 (2020). https://www.state.gov/united-states-strategy-for-central%20-asia-2019-2025-advancing-sovereignty-and-economic-prosperity/. Cited May 21, 2022.

21 Mendkovich, N. (2019) The US and anti-Chinese NGOs in Central Asia, IAC, November 5. https://ia-centr.ru/experts/nikita-mendkovich/ssha-i-antikitayskie-npo-v-tsentralnoy-azii/. Cited January 2, 2022.

22 Ibid.

23 Rodionov, D. (2021) How the West is rocking Kazakhstan and which NGOs will overthrow the government, LIFE, November 9. https://life.ru/p/1448643?ysclid=l3yei65tnw. Cited January 2, 2022.

24 United States strategy for Central Asia 2019‒2025: Advancing sovereignty and economic prosperity (overview) (2022), U.S. Department of State, February 5. https://www.state.gov/united-states-strategy-for-central%20-asia-2019-2025-advancing-sovereignty-and-economic-prosperity/. Cited May 21, 2022.

25 Ibid.
